# Development and Relative Validation of a Food Frequency Questionnaire to Assess Non-Nutritive Sweeteners Intake among Pregnant Women in Santiago, Chile: A Pilot Study

**DOI:** 10.3390/nu15112518

**Published:** 2023-05-29

**Authors:** Sandra López-Arana, Rebeca Peralta, Verónica Sambra, Karen Abrigo, Gabriel Prado, Paola Cáceres, Bielka Carvajal, Martin Gotteland

**Affiliations:** 1 Department of Nutrition, Faculty of Medicine, University of Chile, Av. Independencia 1027, Independencia, Santiago 8380453, Chile; 2 Institute of Nutrition and Food Technology (INTA), University of Chile, Av. El Líbano 5524, Macúl, Santiago 7830490, Chile; 3 Department of Women and Newborn’s Health Promotion, Faculty of Medicine, University of Chile, Av. Independencia 1027, Independencia, Santiago 8380453, Chile

**Keywords:** non-nutritive sweeteners, food frequency questionnaire, survey validation, pregnant women

## Abstract

Studies on the effects of non-nutritive sweeteners (NNSs) among pregnant women are scarce and have produced mixed results. One of the major challenges is to accurately assess NNS intake, especially in countries that have implemented policies to prevent obesity and where many foods and beverages have been progressively reformulated to partially or totally replace sugar with NNSs. This study aimed to develop and assess the relative validity of a food frequency questionnaire (FFQ) for use in pregnant women. We developed an FFQ to examine the intake of seven NNSs (acesulfame-k, aspartame, cyclamate, saccharin, sucralose, steviol glycosides, and D-tagatose). This questionnaire was piloted in 29 pregnant women (median age = 31.2 y; 25th–75th percentile: 26.9–34.7) to assess NNS intake over the previous month, compared to 3-day dietary records (3-DR). The validity of this dietary method was assessed using Spearman’s correlation coefficient, Lin´s concordance correlation coefficient (CCC), and Bland–Altman plots. Spearman’s correlations between the FFQ on NNSs and 3-DR ranged from 0.50 for acesulfame K to 0.83 for saccharin. CCC ranged between 0.22 and 0.66. The Bland–Altman plots showed an overestimation of saccharin, sucralose, and steviol glycosides intake by the FFQ on NNSs compared with 3-DR, and an underestimation of acesulfame K and aspartame. Overall, the NNSs most frequently consumed were sucralose, and none of the participants exceeded the acceptable daily intake for any of the NNSs evaluated. The FFQ on NNSs seems to be reasonably valid in the assessment of NNSs among pregnant women.

## 1. Introduction

Non-nutritive sweeteners (NNSs) are additives used in various foods and beverages to reduce energy density and sugar content, or as tabletop sweeteners [1,2]. They are widely employed to prevent weight gain or facilitate weight loss, or to regulate glucose metabolism in diabetic patients. Due to the growing incidence of obesity and type 2 diabetes, the global consumption of NNSs over the last decade has increased dramatically in adults and children [3,4,5]. In Chile, the law 20.606 on Food Labeling and Advertising sets limits for the sugar, sodium, saturated fat, and total energy content in processed foods [6]. Foods that exceed the limits for one or more critical nutrient must carry a front-of-package warning label stating: “High in …,” with the identity of the corresponding critical nutrient [7]. Since the law came into force, many foods and beverages have been progressively reformulated to replace sugar with NNSs [8,9] and, in consequence, the proportion of processed foods containing NNSs has increased considerably in recent years. We recently showed, for example, that 55.5% of foods available in Chilean supermarkets contained NSSs, which is much higher than in other countries [9].

Alongside the increase in NNS consumption, conflicting results have associated their use with a wide range of adverse health effects related to cancer risk [10], alterations in food intake, mood, blood pressure, body weight, abdominal obesity, and the development of diabetes and neurodegenerative diseases [11,12,13,14]. Accordingly, the benefits of NNS use on human health is the subject of intense debate. In this context, the World Health Organization (WHO) has published a guideline on the use of non-sugar sweeteners which suggests “that non-sugar sweeteners not be used as a means of achieving weight control or reducing the risk of non-communicable diseases (conditional recommendation)” [15].

Of particular interest for the consumption of NNSs is their possible effect on fetal development in utero and their use during pivotal periods such as pregnancy and lactation, due to the possibility of metabolic alterations or other consequences in the mother and offspring [2,3,16,17]. Unfortunately, few studies have been conducted in this area to date. A recent meta-analysis, based on three studies, reported that NNS intake by pregnant women increased the risk of preterm delivery [18]. Another study showed that infants whose mothers consumed NNS during pregnancy and lactation have higher weight gain at one year of age [19]. More studies are therefore necessary to assess the impact of maternal NNS intake on child health.

Finally, the conflicting results reported above regarding the effect of NNS consumption may be partly due to the different approaches used to measure NNS intake. Though the consumption of artificially sweetened beverages is generally used as a proxy for NNS intake [20], the validity of this approach decreases when the variety in foods containing NSSs increases, as in Chile. There is, therefore, an urgent need to develop valid, efficient, and cost-effective instruments to accurately assess NNS consumption. Traditionally, Food Frequency Questionnaires (FFQs) have been the preferred method used in epidemiological studies to assess usual dietary intakes over long periods of time and in large samples, as they are less burdensome, less time consuming, and less intrusive for participants [21,22,23]. It is important to evaluate FFQs in the target population before implementation, and to assess their validity by comparing them to an appropriate dietary reference method [24]. FFQs are usually validated by 24 h recalls or dietary records [25]. To our knowledge, no study has accurately assessed NNS intake in pregnant women, given the variation in NNS content with portion size, brands, and flavors, especially in countries where the number of foods and beverages containing NNSs has dramatically increased [26].

Therefore, the aim of this pilot study was to develop and assess the relative validity of an FFQ to evaluate the intake of NNSs in pregnant women in Santiago, Chile. The NNSs studied were those most commonly found in food and beverages in the Chilean market at the time of data collection [9].

## 2. Material and Methods

### 2.1. Study Design

This work was undertaken as part of the study “Determination of fetal and infant exposure to non-caloric sweeteners study”, whose aim is to determine NNS concentrations (sucralose, saccharin, acesulfame-k, cyclamate, and steviol) in amniotic liquid and breastmilk and correlate them with maternal NNS intake in order to evaluate fetal/infant exposure to these compounds. In addition, it aims to determine whether a higher intake and concentration of NNSs influence sweet taste threshold and preference as well as weight gain during the first year in their offspring (https://clinicaltrials.gov/ct2/show/NCT03972176; accessed on 24 May 2023). The current FFQ on NNSs validation study was conducted as an initial step prior to subject recruitment and data collection for this study. Accordingly, subjects included in the validation step were not participants of the main study.

A convenience sample of pregnant women was recruited from the Santiago Metropolitan Area from May 2019 to September 2019 using flyers, advertisements, social media posts, and word of mouth. These mothers were attended to in Primary Health Care Centers from the surrounding neighborhood area and the Faculty of Medicine at University of Chile. Initially, 75 pregnant women were contacted, and the following eligibility criteria were applied: singleton pregnancy, being in the last trimester of pregnancy, ≥18 years of age, fluent in Spanish, without diagnosis of diabetes prior to pregnancy, and more than two years of residence in Chile. Forty-five women met these criteria and were invited to participate a face-to-face interview. These who agreed to participate signed an informed consent form, completed a short sociodemographic questionnaire, answered some questions regarding their previous pregnancies and current pregnancy, and self-reported their current weight. Trained nutritionists applied the FFQ on NNSs, and upon completion of the face-to-face interview, the women were provided with a three-day dietary record to be returned, once completed, to the nutritionist within the following seven days. The final sample with comprehensive information on the FFQ on NNSS and 3-day dietary records included 29 pregnant women. The Ethics Committee of the Faculty of Medicine at the University of Chile approved the study.

### 2.2. Food Frequency Questionnaire

**Figure 1:**
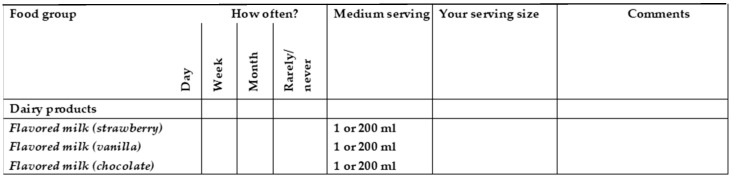
Example of the semiquantitative food frequency questionnaire with open-ended options used in this pilot study.

The development of the FFQ on NNSs was divided into two phases: design (phase 1) and piloted validation (phase 2). In phase 1, a food and beverage list was created specifically to capture NNS intake over the previous month. The NNS content was obtained after trained personnel carried out a comprehensive search visiting nineteen supermarkets and gathering information from thirteen web pages from food, beverage, and tabletop NNSs. The information included ingredients, concentrations per serving size, concentrations per 100 g, brands, and flavors of all foods and beverages available in the Chilean market at the time of data collection [9]. Overall, a total of 815 food and beverage items were found that contained at least one of these NNSs: acesulfame-k, aspartame, cyclamate, saccharin, sucralose, steviol glycosides, and D-tagatose, and they were organized into six food groups: (i) dairy products; (ii) cereal products; (iii) processed fruits; (iv) non-alcoholic beverages; (v) sweets and other desserts; and (vi) tabletop NNSs. All the information was entered in an Excel spreadsheet to assign the values for NNS content in milligrams (mg) to all NNS products found per serving size and per 100 g. Furthermore, to stimulate participants´ memory, a photographic atlas of all the products with NNSs (including specific brands and flavors) ordered by food groups was developed, which also contained images of dishes, glasses, and tablespoons to estimate the size of the ingested portion. The food database and atlas were regularly updated in the course of the study in order to integrate the new NNS-containing products launched on the market and, eventually, to eliminate those that were withdrawn. A semi-quantitative questionnaire with open-ended options was used to prevent the loss of information and misclassification [27] (see Figure 1). Serving sizes were estimated based on amounts commonly purchased or eaten according to the National Chilean Dietary Survey (Encuesta Nacional de Consumo Alimentario, ENCA) [28]. A team of nutritionists reviewed and confirmed the content validity of the questionnaire.

In phase 2, the FFQ information was compiled by a trained nutritionist during the face-to-face interview with the participants. The photographic atlas of all products containing NNS, classified by food groups, was shown to the mothers, who were asked to indicate their usual frequency of consumption of each of them. Next, the serving size was specified in terms of household units of measurement. For each NNS, the amount of daily intake was calculated as follows: Daily NNS intake (mg/day) = [Number of daily serving sizes X Frequency intake monthly X Serving size amount (g or mL) X Concentration of each NNS in the food matrix (mg/100 g)]/30. Afterwards, we determined the daily intake per kilogram of body weight of each NNS by dividing the daily intake previously calculated by the self-reported body weight (mg/kg bw). Finally, such values were summed up to obtain the total daily NNS intake.

### 2.3. Three-Day Dietary Records

A three-day dietary record (3-DR) was used to assess the NNS consumption of all participants over a week. After the face-to-face interview, participants were instructed to provide, as accurately as possible, all foods consumed and highlight those containing NNSs on two weekdays and one weekend day. Records requested information on food and beverage items (with brand names and flavor, where possible), portion sizes in household measures, and time and location of meals. Food records were reviewed, and participants were contacted by the nutritionist to clarify missing or incomplete information. The results were entered into an Excel file to convert all the food/beverage items into a daily NNS intake and the daily intake per kg of body weight record. Intakes from three-day dietary records were averaged across the three days. The Food Processor software 10.9 COPYRIGHT © ESHA RESEARCH, INC., 2011, and the Composition Table of the Chilean Foods, as well as information provided by the food manufacturers, were used to estimate calorie and macronutrient intake.

### 2.4. Statistical Analyses

Statistical analyses were performed with Stata SE version 14 (StataCorp LLC, College Station, TX, USA). Data were checked for normality using the Shapiro–Wilk test and histograms. This analysis determined that continuous variables were not normally distributed; therefore, we report continuous variables as medians and 25th–75th percentile, while or the categorical variables are presented as frequencies and percentages. We described the daily intake of NNSs (mg/day) and the daily NNS intake per kg of body weight per consumer and we compared them with the Acceptable Daily Intake of NNSs (ADI) defined by the European Food Safety Authority (EFSA) [12]. The validity of the FFQ on NNSs was assessed by comparing individual and total NNS intake measured by the FFQ on NNSs against those measured with the average of the 3-DR. Spearman’s correlation was used to identify the relationship between NNS intake obtained from the FFQ on NNSs and the 3-DR. We also analyzed the data using the concordance correlation coefficient by Lin (CCC). It is the correlation between the two measurements that fall on the 45° line through the origin. The CCC ranges from 0, indicating no substantial agreement, up to 1, representing perfect concordance [29]. We preferred CCC over the Intraclass Correlation Coefficient (ICC) or paired t-test because there are some drawbacks to all these methods [29,30,31]. In addition, a sample size calculation was performed for Bland–Altman analysis of agreement [32]. Based on the mean difference between FFQ and Dietary Records as well as standard deviation of the differences from Myers at al. [20], a maximum allowed difference of 80 mg among total NNSs, an α level of 0.05, and a power of 0.8, a sample size of 25 was required. The Bland–Altman plot [33] was used to graphically assess the agreement between both instruments using mean difference and 95% limits of agreement (LoA). The analyses of the CCC and the Bland–Altman plots were conducted using the Stata “concord” command. The results were considered statistically significant at a 0.05 level (two-tailed).

## 3. Results

### 3.1. Descriptive Statistics

A total of 45 women were recruited, but only 29 completed the study. The mean of the participants was 31.2 years (25th–75th percentile: 26.9–34.7), and 69% were primiparous and with a median of 30 gestational weeks. More than two thirds of the sample were Chilean, with the remainder being of other nationalities, mainly Venezuelan, Peruvian, Colombian, and Ecuadorian. Their average daily energy intake was 1873 ± 416 kcal. More than half (52.7%) of the total calorie intake came from carbohydrates, while total fats and proteins contributed 32.3% and 15%, respectively. More than 65% of the participants had a high level of education and 80% were in the labor force (Table 1).

Of the 29 participants, 93.1% (*n* = 27) consumed NNSs daily in the FFQ, while 82.8% (*n* = 24) consumed NNSs in the 3-DR. The three NNSs most frequently consumed were sucralose, followed by steviol glycosides and acesulfame K. Overall, the median daily NNS intakes of sucralose, steviol glycosides, and D-tagatose according to the FFQ were higher than the intakes reported through the 3-DR. The median daily NNS intake from the FFQ on NNSs was 1.49 mg/kg bw (25th–75th percentile: 0.73–3.37) vs. 1.28 mg/kg bw (25th–75th percentile: 0.62–2.48) daily NNS intake reported from the 3-DR. When compared with ADI, none of the participants exceeded the acceptable daily intake for any of the NNSs evaluated (Table 2).

### 3.2. Correlations

Table 3 shows the correlations of the estimates of NNSs and total NNS intake between both methods. Correlations for each of the seven NNSs and total NNS intake were statistically significant (*p <* 0.05). Spearman’s correlation coefficients were moderate to strong and ranged from 0.50 for acesulfame K to 0.83 for saccharin. In the case of cyclamate and D-tagatose, the correlations were not applied due to the very small sample that consumed those NNSs. The Lin´s CCC was used to assess the reliability between FFQ on NNSs and the 3-DR. Within the seven NNSs and total NNSs assessed, we found a moderate level of agreement for saccharin (CCC = 0.66; 95% CI: 0.58 to 0.74), while for aspartame, there was not agreement (*p >* 0.05). This suggests that although the values of both methods are associated, the level of agreement was much poorer.

### 3.3. Agreement between Methods

**Figure 2:**
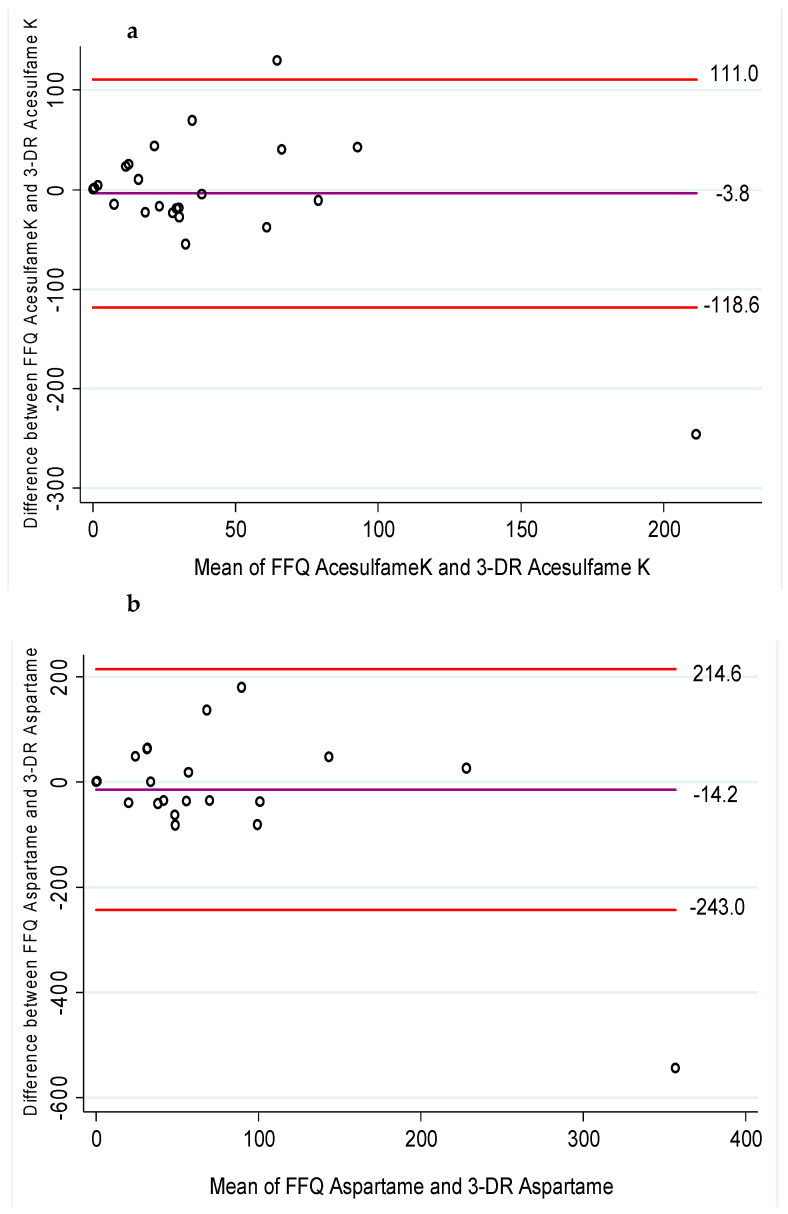

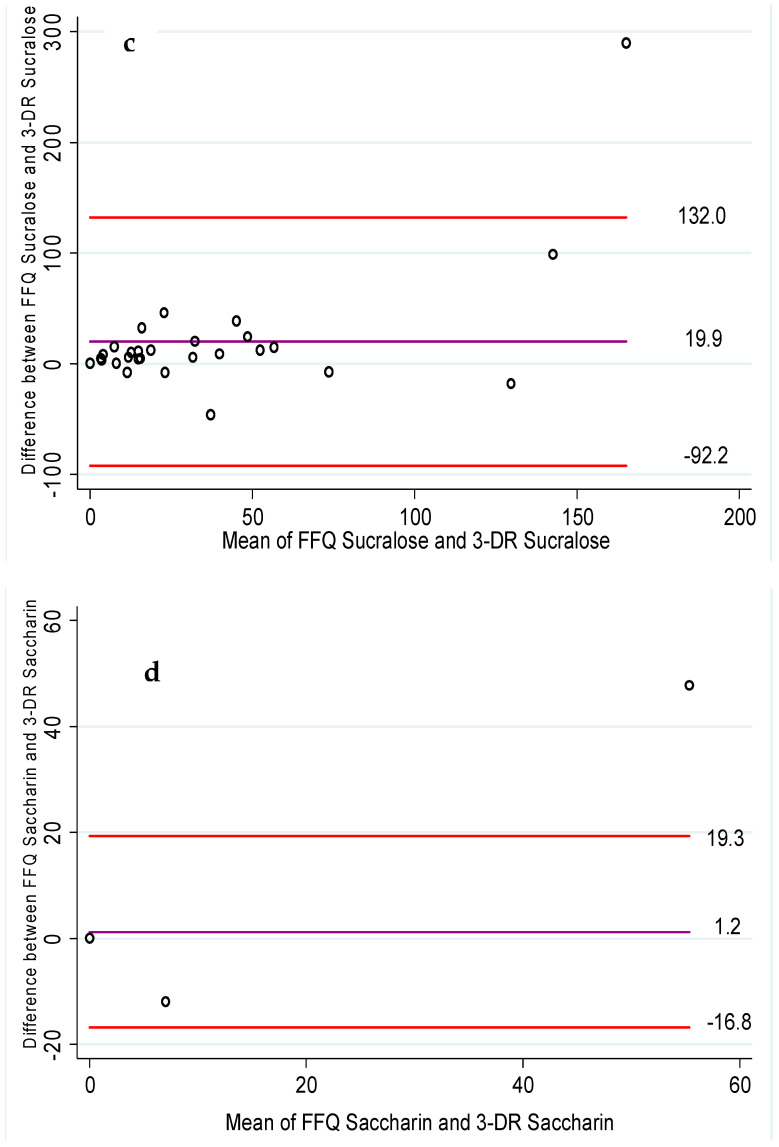

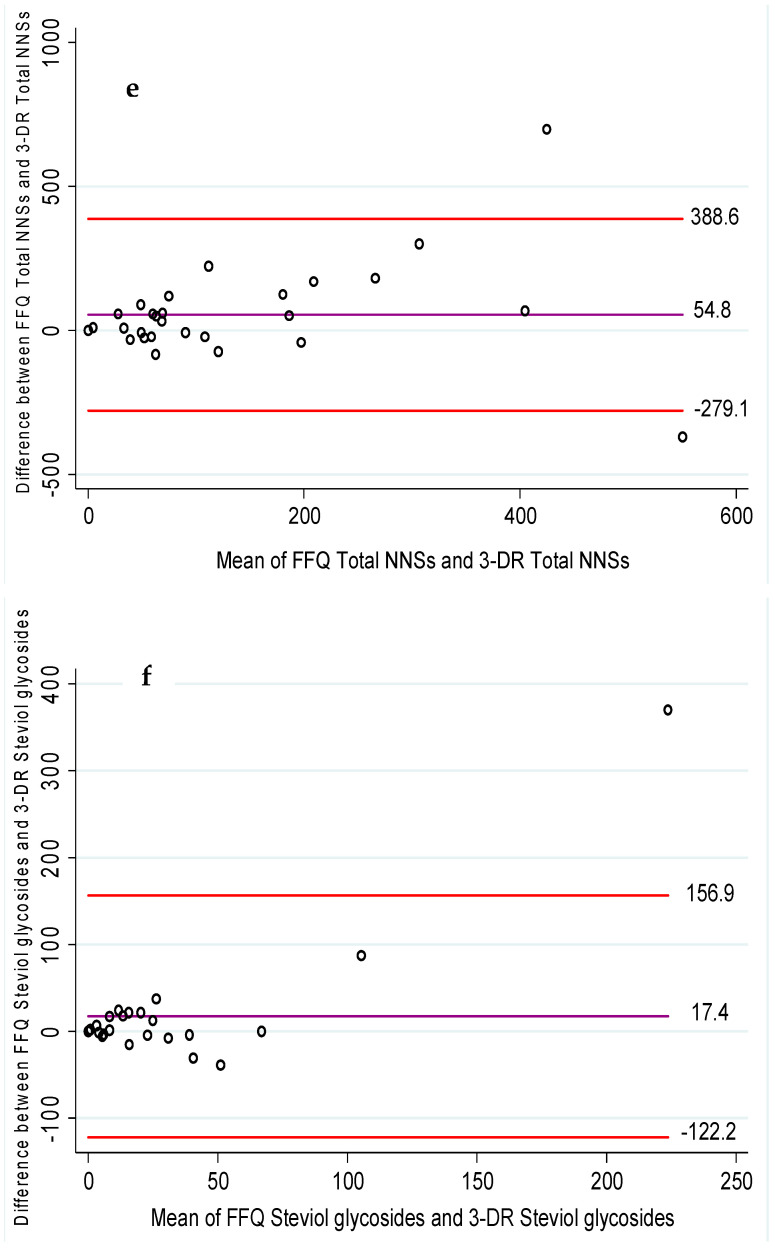
Bland–Altman plots assessing the agreement of daily NNS intake (mg/day) from the FFQ on NNSs and the average of the 3-DR. The center line represents the mean difference between the two methods and the upper and lower lines represent the 95% LoA for (**a**) acesulfame K; (**b**) aspartame; (**c**) saccharin; (**d**) sucralose; (**e**) steviol glycosides; and (**f**) total NNS intake. Bland–Altman plots (…/…).

The Bland–Altman plots (Figure 2) measure the agreement of mg intake of total and each type of NNS between the FFQ and 3-DR. Acesulfame K and aspartame were underestimated in the FFQ compared to the 3-DR, while other NNSs and total NNSs were overestimated. The mean differences in daily NNS intake were: −3.8 mg/day for acesulfame K, −14.2 mg/day for aspartame, 1.2 mg/day for saccharin, 19.9 mg/day for sucralose, 17.4 mg/day for steviol glycosides, and 54.8 mg/day for total NNSs.

## 4. Discussion

In this pilot study, we developed and assessed the validity of an FFQ to evaluate the intake of seven NNSs in pregnant women living in Santiago, Chile. To our knowledge, this is the first study to demonstrate the validity of an FFQ using a list of most food products containing NNSs available in the local market after the Chilean law on food labeling and advertising came into force, and the subsequent reformulation of food and beverage products. Our results indicate that the FFQ on NNSs had fair-to-moderate validity. The Spearman’s correlation between the FFQ on NNSs and 3-DR was >0.50 for most NNSs; three of them were above 0.60. For cyclamate and D-tagatose, we preferred to exclude the correlations to avoid a false sense of relationship as a large number of participants did not consume these NNSs [34]. This is because only 1.3% of foods containing NSSs in the Chilean market contain cyclamate [9]. In the case of D-tagatose, Article 146 of the Chilean food sanitary regulations states that the use of D-tagatose as an NNS is not allowed in foods intended for weight control diets, nor in foods that are sugar-free, or low or reduced in sugar or sugars (mono- and disaccharides), calories, and fat [35]. While tagatose was originally evaluated as a food additive, it is now considered a novel food in some places such as the United States, Australia, New Zealand, and the European Union [36].

A similar correlation was observed by Myers et al. [20], who measured five types of NNSs (saccharin, aspartame, acesulfame K, sucralose, and erythritol) in adults residing in southwest Virginia. The Spearman’s correlation between the FFQ and three 24 h dietary records ranged from 0.51 to 0.59 [20]. Although a direct comparison is not possible, due to differences in food classification and methods of assessing NNS consumption, similar correlations were observed for acesulfame K and aspartame. In addition, we observed higher correlations for saccharine and sucralose in our study. Overall, these values are similar to those reported in FFQ validation studies, which generally consider correlations between 0.4 and 0.7 to be valid [24,37].

Historically, the most common method for testing the validity of FFQs is the ICC. However, the ICC is very sensitive to sample heterogeneity [38]. We incorporated Lin’s concordance coefficients to overcome these criticisms for assessing agreement for both methods and capitalizing that the “concord” command in Stata also provides Bland and Altman’s limits of agreement graphics [39]. Our results indicate a fair to moderate agreement, but with a proportional bias, i.e., where the differences between the methods tend to first reduce and then increase when measuring larger NNS intakes. The overall mean differences in total NNSs indicate that the FFQ on NNSs tends to overestimate the total intake by 55 mg, which would be equivalent to 138 mL of diet soda. In the Chilean market, 100 mL of diet soda contains 24 mg of aspartame and 16 mg of acesulfame K. Moreover, we found an overestimation of saccharin, sucralose, and steviol glycosides intake by the FFQ on NNSs compared with the 3-DR, and an underestimation of acesulfame K and aspartame. These mean differences were larger than those reported by Myers et al., especially for sucralose and aspartame [20].

Previous studies that assessed NNS consumption in Chile mainly focused on children [40,41,42,43] and adults [44,45] generally, and only one study has addressed pregnant women [46]. To evaluate NNS intake, different dietary instruments have been used such as the 24 hours’ recall [43] or the FFQ [40,42,44,46]. The studies that used FFQ have some shortcomings. Durán et al. designed an FFQ based on a list of 122 food products available in supermarkets. Nonetheless, it was not clear what type of validation was performed [40]. Similarly, Fuentealba et al. pointed out that their FFQ was validated by experts, but without further details [46]. On the other hand, Martínez et al. assessed NNS intake among 250 Chilean children aged 6–12 years using an FFQ originally designed for the US population [42]. Because of differences in dietary habits, the use of dietary instruments in other populations may not be an appropriate alternative and caution should be taken when interpreting the results [24,37]. In addition, the use of methods with limited validity may produce a regression dilution bias [47] that will lead to a serious underestimation of the associations between intake and disease in epidemiological studies [48].

Our results show that, with both dietary instruments, more than three quarters of pregnant women consumed NNSs daily, mainly sucralose, and none of them exceeded the acceptable daily intake for any of the NNSs assessed. This high prevalence of pregnant women reporting their intentional intake of NNSs may be of concern. There is increasing evidence to suggest that pregnant women consume as much or even more NNS as the general population [3]. This steady increase in consumption is probably due to increased awareness of the harmful effects of excessive sugar consumption [16], but also to the massive presence of NNSs in different food products. Indeed, we recently found that out of 1489 products available on the Chilean market, more than fifty percent contained at least one NNS, which is a particularly high proportion when compared to other countries [9]. However, few studies have been conducted on humans to elucidate the effects of NNS consumption during pregnancy and the postnatal period, as well as the long-term consequences on the offspring.

The strengths of this study are that the FFQ on NNSs is a dietary instrument that allows for the quick and easy measurement of NNS consumption. In addition, it can differentiate between sources of NNSs, it is brand specific, and it captures both beverage and food sources of NNSs, since the FFQ on NNSs was developed considering all possible sources of NNSs in the Chilean market. Nevertheless, our study has several limitations. Although more than one third of the Chilean population lives in Santiago, according to the National Statistics Institute (INE), this study was a convenience sample. Therefore, the participants’ backgrounds do not necessarily reflect the pregnant women living in the country. Secondly, the education level of the participants in this study was higher than that of the general population [49]. Nevertheless, a higher level of education was associated with a higher accuracy of responses to the FFQ and 3-DR [50]. As Chile is increasingly receiving immigrants, especially from Latin America and Caribbean countries, with the estimated number of foreign residents reaching 1,492,522 (approximately 10% of the population) [51], we included pregnant women from other nationalities such as Venezuela, Peru, Colombia and Ecuador. However, regional food and beverage items were not taken into account, so the FFQ likely underestimates NNS intake. In addition, our sample size was insufficient to conduct a sub-group analysis.

## 5. Conclusions

In conclusion, this pilot study demonstrated that, at least for the assessment of the consumption of some NNSs, the FFQ can be used for pregnant women living in Santiago, Chile. The FFQ on NNSs may be a useful method for future epidemiological studies on the nutritional status of pregnant women. However, it would be advisable to also consider biological markers as a reference method for future validation studies and to assess the validity of this FFQ in multiethnic populations.

## Figures and Tables

**Table 1:** Characteristics of participants in the pilot study *n* = 29.

Characteristics	25th–75th Percentile or %
Age (years), median (IQR) ^1^	31.2 (26.9–34.7)
Gestational weeks, median (IQR) ^1^	30.0 (28.0–32.0)
Parity *n* (%)	
0	20 (69.0)
≥1	9 (31.0)
Pre-gestational weight, median (IQR) ^1^	60.0 (55.0–66.0)
Current weight, median (IQR) ^1^	68.0 (63.0–73.0)
Medical history *n* (%)	
Gestational diabetes	3 (10.3)
Hypothyroidism	3 (10.3)
High blood pressure	1 (3.4)
Nationality *n* (%)	
Chilean	20 (69.0)
Other	9 (31.0)
Education *n* (%)	
High school	7 (24.1)
Vocational training	3 (10.4)
College or more	19 (65.5)
Employment *n* (%)	
Yes	23 (79.3)
No	6 (20.7)
Diet	
Total energy intake (kcal/d). Mean ± SD ^2^	1873 ± 416
Protein intake (g/d). Mean ± SD ^2^	75.5 ± 26.3
Carbohydrate intake (g/d). Mean ± SD ^2^	245.3 ± 64.0
Fat intake (g/d). Mean ± SD ^2^	70.0 ± 7.5

^1^ IQR: Interquartile range. ^2^ SD: Standard deviation.

**Table 2:** Daily NNS intake (mg/day) and daily NNS intake per kilogram of body weight (mg/kg bw) from the FFQ on NNSs and the average of the 3-DR, and comparison with the Acceptable Daily Intake (ADI) by EFSA among pregnant women reporting any consumption of NNSs.

NNS Type	*n* ^a^	FFQ on Daily NNS Intake (mg/day)	FFQ on Daily NNS Intake (mg/kg bw)	*n* ^a^	3-DR of Daily NNS Intake (mg/day)	3-DR of Daily NNS Intake (mg/kg bw)	ADI Defined by EFSA (mg/kg bw)
		Median (25th–75th Percentile)	Median (25th–75th Percentile)		Median (25th–75th Percentile)	Median (25th–75th Percentile)	
Acesulfame K	23	20.9 (7.0–69.7)	0.38 (0.11–1.00)	15	40.5 (32.0–71.6)	0.65 (0.45–1.16)	9
Aspartame	19	59.1 (24.0–84.6)	0.38 (0.02–0.86)	14	84.0 (58.5–120.0)	0.40 (0.01–0.93)	40
Cyclamate	1	3.1 ^b^	0.06 ^b^	1	38.5 ^b^	0.71 ^b^	7
Saccharin	3	1.1 (0.01–79.2)	0.02 (0.00–1.28)	2	22.3 (13.0–31.5)	0.37 (0.24–0.51)	5
Sucralose	27	24.8 (14.6–60.6)	0.38 (0.20–0.94)	23	22.4 (9.4–46.5)	0.33 (0.17–0.75)	15
Steviol glycosides	25	22.0 (4.2–31.2)	0.31 (0.06–0.49)	18	21.6 (7.8–41.3)	0.31 (0.12–0.62)	4
D-Tagatose	1	3.5 ^b^	0.05 ^b^	1	2.7	0.04 ^b^	not specified
Total NNS intake	27	93.0 (47.6–242.7)	1.49 (0.73–3.37)	24	68.0 (36.7–104.8)	1.28 (0.62–2.48)	not specified

^a^ *n* is the number of participants that consumed any NNSs. ^b^ The 25th and 75th percentile could not be calculated because many participants did not consume these NNSs.

**Table 3:** Spearman’s correlation coefficient (rho) and Lin´s concordance correlation coefficient (CCC) between daily NNS intake per kilogram of body weight and the average of the 3-DR among pregnant women.

NNS Type	Rho Coefficients between FFQ and 3-DR	CCC between FFQ and 3-DR (95% CI)
Acesulfame K	0.50 *	0.37 (0.07–0.61) *
Aspartame	0.51 *	0.28 (−0.01–0.52)
Cyclamate	n/a ***	n/a ***
Saccharin	0.83 **	0.66 (0.58–0.74) **
Sucralose	0.67 **	0.35 (0.09–0.56) **
Steviol glycosides	0.79 *	0.22 (0.04–0.38)
D-Tagatose	n/a ***	n/a ***
Total NNS intake	0.66 **	0.39 (0.02–0.66) *

* *p* ≤ 0.05. ** *p* ≤ 0.01. *** n/a = not applied.

## Data Availability

The atlas and the corresponding Excel data sheet are available on request.
